# Current and Voltage Measurements in the Gaseous Electronics Conference RF Reference Cell

**DOI:** 10.6028/jres.100.026

**Published:** 1995

**Authors:** Mark A. Sobolewski

**Affiliations:** National Institute of Standards and Technology, Gaithersburg, MD 20899-0001

**Keywords:** current, diagnostic, discharge, electrical, Gaseous Electronics Conference, impedance, plasma, radio frequency, voltage

## Abstract

Measurements of the electrical characteristics of discharges in the Gaseous Electronics Conference Radio-Frequency Reference Cell are reviewed here. Topics include: common sources of error in the measurements; comparisons of current and voltage data among GEC cells; the effects of gas impurities, surface conditions and the external circuitry on the reproducibility of the electrical characteristics; and comparisons of current and voltage data with results of other measurements.

## 1. Introduction

Of all the measurements performed on the GEC cell, measurement of the discharge current and voltage are perhaps the most common. Because the current-voltage characteristics are sensitive to a wide variety of plasma properties and perturbations, and because the equipment necessary to measure them is relatively flexible and inexpensive, current-voltage measurements were proposed, quite soon after the construction of the first GEC cells, as a practical means of gauging the reproducibility of plasma conditions among the cells. Electrical characteristics measured in many different cells have been compared [1,2], and the results of these comparisons will be briefly reviewed here. In general, good agreement between cells was obtained, but only after careful consideration and elimination of measurement errors. Some sources of error can be quite large—large enough to invalidate comparisons between cells. Therefore, some discussion is included here on the methods used to eliminate or minimize these errors. In addition, controlled experiments performed in single cells have been very useful in identifying several sources of irreproducibility in plasma conditions. These experiments will also be reviewed. Finally, without any means of interpreting the electrical characteristics, it is difficult to know whether a measured change in electrical data represents an important change in plasma conditions. This paper will therefore also review a limited number of experiments that compare electrical measurements with the results of other diagnostic techniques to yield some insight into the origin of the electrical characteristics and aid in their interpretation.

## 2. Measurement Issues

### 2.1 Probe Accuracy

Current and voltage characteristics of discharges in the GEC cell have been measured using digital oscilloscopes equipped with a variety of probes, including home-made capacitive voltage probes and inductive d*I*/d*t* current probes [1,3] and commercially-available current transformers and attenuating voltage probes [1]. Any of these probes, or the oscilloscope itself, can be an important source of systematic errors. Thus, detailed consideration of these errors and careful implementation of calibration procedures are necessary. Both magnitude and phase errors can be significant. Phase errors arise primarily from propagation delays in the probes and in the cables that connect them to the oscilloscope. The phase error of a voltage probe can be determined from a direct measurement of its delay, using two channels of the oscilloscope. The relative delay between current and voltage probes, and the resulting error in impedance phase, can be determined by attaching the probes to loads of known impedance phase. This can be achieved particularly conveniently using the parasitic impedance of the cell itself as a load [1–3]. If both inductive and capacitive loads are used, it is possible to distinguish true propagation delays from small uncertainties in phase that arise if either load contains an unknown resistance [4].

Another possible source of phase errors is crosstalk between the current and voltage signals. Crosstalk can occur internally in the oscilloscope or in commercial current probes that are improperly grounded [4]. Because of capacitive coupling between the power lead and the case of a current probe, a spurious signal in phase with the voltage can be added to the current signal, shifting its phase. Phase errors of this sort as large as 10° have been detected. The error is minimized by using current probes with higher gain (more volts per ampere) and by assuring a good connection between the case of the current probe and the oscilloscope ground. Large errors will, of course, also occur if the current probe is installed with the wrong polarity or if its output is improperly terminated.

Systematic errors in amplitude measurements can also be large, especially at high frequencies near the bandwidth limits of the probes or the oscilloscope. Bandwidths are usually specified by a 3 dB frequency. At this frequency amplitudes are in error by a factor of $\surd \bar{2}$. Even at frequencies far below this, bandwidth limitations degrade measurement accuracy; often the effects of limited bandwidth become negligible only at a frequency one power of ten below the 3 dB frequency. Many probes and oscilloscopes with bandwidth sufficient to permit excellent accuracy at 13.56 MHz are available and relatively inexpensive. Unfortunately, it is more difficult to obtain accurate measurement of the higher frequency harmonic signals generated by the plasma. There also appears to be a tradeoff between the bandwidth of probes and the maximum current or voltage that they can tolerate. These problems can be solved by calibrating probe amplitudes against higher bandwidth instruments or by constructing capacitive voltage probes and inductive d*I/*d*t* current probes [1,3]. These have extremely high bandwidths, and have the added advantage of amplifying the weak harmonic signals.

### 2.2 Cell Parasitics

At radio-frequencies, the GEC cell contains significant parasitic impedances, including stray capacitance, self-inductance and parasitic series resistance. Measured current and voltage waveforms include contributions from the parasitics as well as the plasma. Furthermore, the exact value of the parasitics can be quite sensitive to minor changes in the design of the electrodes and small shifts in the positions of the probes. If the values of the parasitics vary from cell to cell, probe measurements will vary, even when plasma conditions are identical. In this situation, procedures are required to convert the current and voltage waveforms measured by the probes into waveforms more indicative of the plasma itself: waveforms representing the current and voltage present inside the cell, at surfaces in contact with the plasma. This section describes procedures that characterize and correct for the parasitics. It should be noted that the parasitics are also important for another reason: they, together with the remainder of the external circuitry that powers the cell, establish boundary conditions on the plasma, and variations in these boundary conditions can cause real variations in plasma electrical characteristics. This topic will be discussed separately in a later section.

**Fig. 1:**
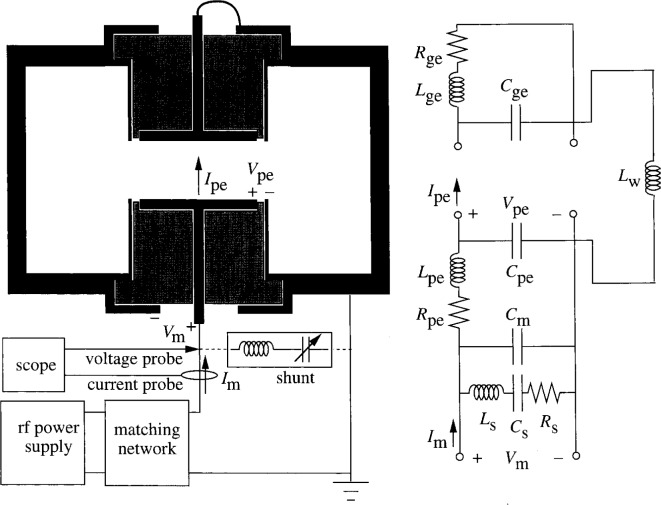
Equivalent circuit diagram of the GEC cell. Shown on the left is a block diagram for the cell, operated with one electrode powered and one electrode grounded, with a shunt circuit attached. The equivalent circuit for this configuration is shown on the right. The circuit includes parasitics in the ground electrode assembly (*C*_ge_, *L*_ge_, *R*_ge_), in the powered electrode assembly (*C*_pe_, *L*_pe_, *R*_pe_, *C*_m_), in the chamber walls (*L*_w_) and in the shunt circuit (*L*_s_, *C*_s_, *R*_s_).

Parasitics in the GEC cell have been characterized at 1 MHz to 100 MHz using a vector impedance meter [5] and over a narrower frequency range using current and voltage probes [4,6]. From these studies the equivalent circuit model of the parasitics shown in Fig. 1 was obtained. This model represents the cell in its most common mode of operation: with one electrode powered and the other grounded, and a shunt circuit [1,2,7] attached. The terminals at the bottom of the circuit diagram represent the point on the powered electrode lead at which the current and voltage probes are mounted. The current and voltage measured by the probes, *I_m_*(*t*) and *V_m_*(*t*), are defined in Fig. 1, as are *I*_pe_(*t*) and *V*_pe_(*t*), the current and voltage at the surface of the powered electrode. The equivalent circuit diagram shows all cell parasitics, including both the powered and ground electrode. (Parasitics in the electrical network upstream of the probes are not shown.) The parasitic capacitance *C*_pe_ is largely associated with the thin sleeve of insulator between the powered electrode and its ground shield. The long lead that powers the powered electrode and the insulator and ground shield that surround it act as a transmission line that contributes most of the inductance *L*_pe_ and resistance *R*_pe_, and part of the capacitances *C*_pe_ and *C*_m_. *C*_m_ also includes the parasitic capacitance of the current and voltage probes and their supports. Similarly, the parasitics *L*_ge_, *C*_ge_ and *R*_ge_ are associated with the upper, grounded electrode. No capacitance analogous to *C*_m_ is shown for the upper electrode, as the ground connection for this electrode short-circuits any such capacitance. *L*_w_ represents the self-inductance of the cavity between the chamber wall and the outer surface of the ground shields. *L*_s_, *C*_s_ and *R*_s_ represent the shunt circuit [1,2,7] which consists of a coil and an air-variable capacitor connected between the power lead and the chamber ground, just downstream from the current probe. The shunt is designed so that, at the fundamental frequency of 13.56 MHz, it has an inductive impedance that cancels the net capacitive reactance of the rest of the cell, thereby reducing the total current drawn by the cell, improving the precision of current measurements [4], and alleviating problems with rf interference, ground loops, and current probe overload.

The exact values of the parasitic elements shown in Fig. 1 vary from cell to cell. The capacitance values depend on the insulator material—alumina or Teflon^1^. Teflon insulators have lower capacitances due to their smaller dielectric constant [1]. For alumina insulators, two designs exist, the original design having a solid core and an updated, more easily fabricated hollow-core version with slightly lower capacitance values. Finally, some random variation in *C*_pe_ and *C*_ge_ are exhibited between cells, as these capacitances can be very sensitive to the exact dimensions of the insulator and its alignment relative to the electrode and ground shield. The self-inductance *L*_pe_ also varies between cells, depending on how far away the probes are installed from the powered electrode. *C*_m_ depends on the particular probes used, and the shunt’s parameters vary according to the details of its construction. Despite these variations, it is believed that the circuit model of Fig. 1 is general enough to accommodate any GEC cell in the standard configuration. Of course, cells that have been drastically modified to incorporate alternative sources, mass spectrometers, or topside optical access will have very different equivalent circuits.

Because of the variations in parasitics from cell to cell, electrical data are best expressed in terms of *I*_pe_(*t*) and *V*_pe_(*t*), the current and voltage at the surface of the powered electrode, rather than *I*_m_(*t*) and *V*_m_(*t*), the current and voltage measured by the probes. To achieve this, an equivalent circuit for the cell is typically assumed, a limited set of measurements are performed with the plasma extinguished to determine values for the parasitics, and the circuit equations are then solved to obtain *I*_pe_(*t*) and *V*_pe_(*t*). The model most commonly used [1,2,7] includes four elements (*C*_pe_, *L*_pe_, *C*_s_ and *L*_s_) but it omits *C*_m_, *R*_pe_ and *R*_s_. Accurate values of $I_{{\text{pe}}_{1}}$ and $V_{{\text{pe}}_{1}}$, the fundamental components of *I*_pe_(*t*) and *V*_pe_(*t*), can be obtained from this model, if care is taken to match the model to measured characteristics, and if the capacitance of the gap between the electrodes is properly accounted for. (Otherwise, if the gap capacitance is included in *C*_pe_, systematic errors in $I_{{\text{pe}}_{1}}$ as large as 15 % are possible [4]). However, the omission of resistive parasitics, particularly *R*_s_, can introduce large systematic errors in *θ*, the phase between $V_{{\text{pe}}_{1}}$, and $I_{{\text{pe}}_{1}}$, and in the plasma power $P_{\text{pe}}=1/2\;I_{{\text{pe}}_{1}}\;V_{{\text{pe}}_{1}}\cos\;\theta$. Indeed, the simple four-element model yields values of *θ* and *P*_pe_ that differ by as much as 6° and 60 %, respectively, from values obtained using a general treatment [4] that includes the resistive parasitics.

The procedures that account for the parasitics (and for probe phase errors) are conveniently performed in the frequency domain. Therefore, the first step in the analysis of measured waveforms is almost always Fourier analysis, most commonly accomplished using the Fast Fourier transform (FFT) algorithm. Applying the FFT directly on the digitized waveforms does not yield the desired Fourier coefficients exactly, however, because in general the sampling rate and the rf frequency are not commensurate. Instead, linear interpolation between the measured data points is used to generate a waveform with an appropriate time-base and spacing between points. Applying the FFT to the interpolated waveform then yields the Fourier coefficients at the exact frequencies of the fundamental and harmonics, free from aliasing effects. Alternatively, Fourier analysis can be accomplished using curve-fitting techniques [8] or by explicit evaluation of the Fourier integrals [6]. These methods can be very efficient, more efficient than the FFT, as no time is wasted calculating the amplitudes of components known to be insignificant. Analysis methods have recently been extended to include transient effects [9].

## 3. Cell Comparisons

**Fig. 2:**
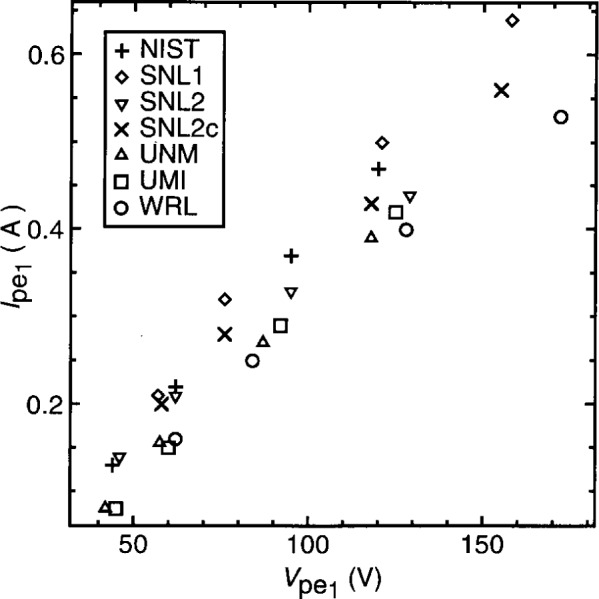
Comparison of $I_{{\text{pe}}_{1}}$ and $V_{{\text{pe}}_{1}}$ the fundamental amplitudes of current and voltage at the surface of the powered electrode, for argon discharges at 66 Pa (500 mTorr) in six different GEC cells. (In one cell, SNL2, measurements were performed twice, with and without added capacitance.) Data from Ref. [1].

**Fig. 3:**
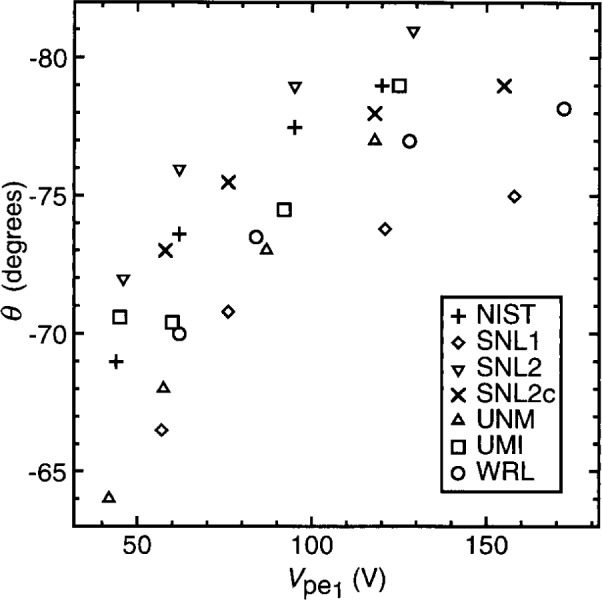
Comparison of electrical data from six GEC cells. The phase *θ* between $I_{{\text{pe}}_{1}}$ and $V_{{\text{pe}}_{1}}$ the fundamental components of current and voltage at the surface of the powered electrode, is plotted against the magnitude of $V_{{\text{pe}}_{1}}$, for argon discharges at 66 Pa (500 mTorr). Data from Ref. [1].

**Fig. 4:**
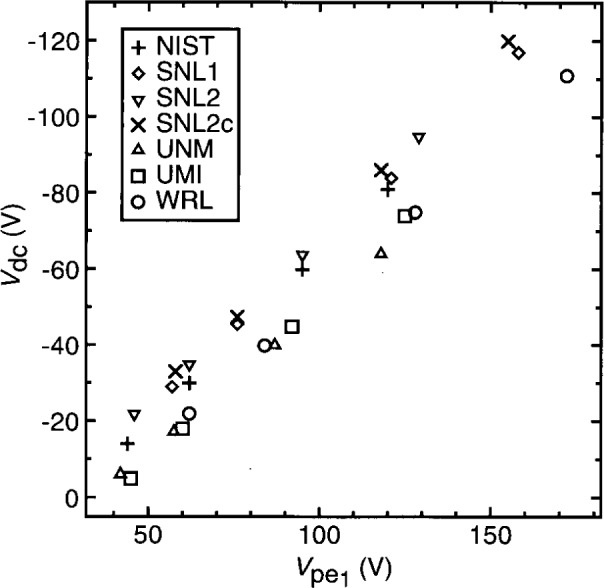
Comparison of electrical data from six GEC cells. The dc component *V*_dc_ of the voltage on the powered electrode is plotted against the fundamental component $V_{{\text{pe}}_{1}}$, for argon discharges at 66 Pa (500 mTorr). Data from Ref. [1].

To assess the reproducibility of plasma conditions among GEC cells, the current and voltage characteristics of argon discharges in may cells, under nominally identical conditions, have been measured and compared [1,2]. In one study, measurements were made in six cells at five different laboratories, at four values of pressure and four values of applied voltage [1]. Figures 2 to 4 show results of this comparison at a pressure of 66 Pa (500 mTorr). The fundamental components $I_{{\text{pe}}_{1}}$ and $V_{{\text{pe}}_{1}}$ of the corrected current and voltage waveforms *I*_pe_(*t*) and *V*_pe_(*t*) are plotted in Fig. 2. In Fig. 3 the phase difference *θ* between $I_{{\text{pe}}_{1}}$ and $V_{{\text{pe}}_{1}}$ is plotted, and Fig. 4 shows the dc self-bias *V*_dc_. In each cell data were measured at peak-to-peak applied voltages of 75 V, 100 V, 150 V, and 200 V. Because the parasitics in the cells differ, identical values of applied voltage, as measured by the voltage probe, do not correspond to identical values of $V_{{\text{pe}}_{1}}$. Thus the *x*-axis values for the different cells are not identical. Data from cells with higher values of the parasitics *L*_pe_ and *C*_pe_ appear shifted to the right, while cells with lower values of *L*_pe_ and *C*_pe_ appear shifted to the left.

Nevertheless, shifts in the $V_{{\text{pe}}_{1}}$ values are accompanied by shifts in $I_{{\text{pe}}_{1}}$, so that the data points plotted in Fig. 2 fall roughly on the same line. This illustrates that a reasonable degree of reproducibility can be obtained between cells, even though the parasitics differ, by operating the cells at identical values of $V_{{\text{pe}}_{1}}$. Because the parasitics differ, this parameter, rather than as-measured voltages, is a more useful way of specifying and reporting the operating point of the plasma. Figures 3 and 4 show that reasonable agreement in θ and *V*_dc_ can also be obtained when different cells are operated at the same value of $V_{{\text{pe}}_{1}}$.

It is difficult to interpret the variance between cells seen in Figs. 2 to 4. Some part of it is undoubtedly due to measurement error. The probes and oscilloscopes used to acquire the data have accuracy specifications of only a few per cent. For some probes, with upper 3 dB frequencies close to 13.56 MHz, larger errors are expected. Furthermore, errors in the models and measurements used to characterize the parasitics can produce errors in the calculated values of $I_{{\text{pe}}_{1}}$, $V_{{\text{pe}}_{1}}$ and *θ.* This is particularly true of *θ.* The values of *θ* shown in Fig. 3 were calculated without considering resistive parasitics in the cell and the shunt. This produces a systematic error in *θ* which, in the NIST cell, is typically 5° [4]. Other cells will have systematic phase errors that are similar, but not identical, thus contributing to the variations between cells seen in Fig. 3.

Alternatively, some of the observed variance is *not* due to measurement errors, but to true variations among the cells. There are a number of mechanisms by which these variations could arise. The discharge is affected by the condition of electrode surfaces, the concentration of gas-phase impurities, and the electrical boundary conditions imposed on the discharge by the cell and the external circuit that powers it. Differences in any of these properties could be in part responsible for the deviations seen in Figs. 2 to 4. To elucidate the role of each of these mechanisms, however, cell comparisons are of limited usefulness. Instead, controlled experiments in single cells, as described in following sections, have been performed to investigate each of these phenomena individually.

## 4. Plasma Reproducibility Issues

### 4.1 External Circuit Effects

In general, the electrical characteristics of rf discharges are nonlinear. Therefore, when driven at one frequency, a discharge generates signals at harmonic frequencies that propagate back through the external circuitry that powers the cell. The magnitudes and phases of the harmonics depend on the external circuit. Indeed, the ratio of a pair of voltage and current harmonics equals the impedance of the external circuit at the harmonic frequency, looking back upstream from the measurement point towards the power supply (provided that harmonics are generated only by the discharge). Therefore, if the impedance of the external circuit changes, the harmonic signals must also change. Again, because the discharge is nonlinear, the resulting changes need not be confined to the harmonics; the fundamental current and voltage components and the dc self-bias may also change.

Sensitivity of the electrical characteristics to the external circuitry has been observed in cell comparisons. For example, for the cells compared in Figs. 2 to 4, harmonic components differed widely [1]. This is expected, as the external circuits of the cells were not standardized. Agreement in the harmonics was obtained only for one pair of cells, in which special care had been taken to make sure that the cell parasitics, the power supply and matching network, and the rest of the external network were electrically identical [1].

**Fig. 5:**
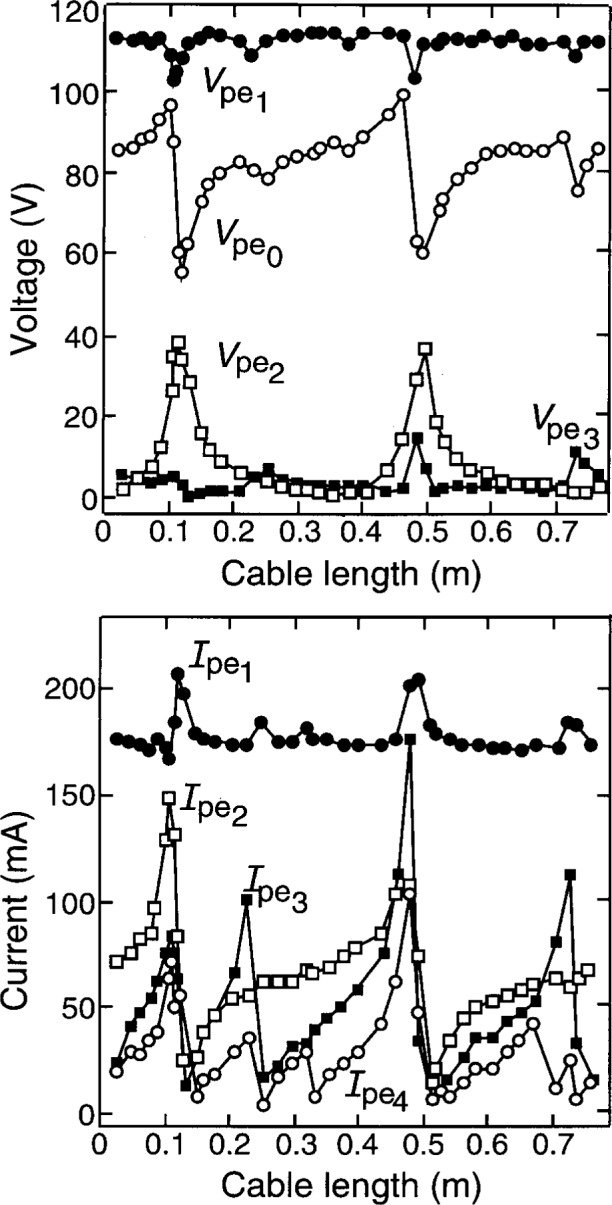
Amplitudes of the harmonic components of the voltage and current waveforms on the powered electrode of the GEC cell, as a function of the length of a cable that powers the cell, for an argon discharge at 13.3 Pa (100 mTorr). The fundamental (13.56 MHz) components $V_{{\text{pe}}_{1}}$ and $I_{{\text{pe}}_{1}}$ are shown, along with the dc component of voltage $V_{{\text{pe}}_{0}}\equiv V_{\text{dc}}$, components $V_{{\text{pe}}_{2}}$ and $I_{{\text{pe}}_{2}}$ at 27.12 MHz, $V_{{\text{pe}}_{3}}$ and $I_{{\text{pe}}_{3}}$ at 40.68 MHz, and $I_{{\text{pe}}_{4}}$ at 54.24 MHz. Data from Ref. [3].

In one cell, external circuit effects were studied systematically, by varying the length of the cable between the cell and the matching network [3]. As the cable length is varied, its impedance changes cyclically, with a periodicity determined by the wavelength. Figure 5 shows how the corrected current and voltage amplitudes responded to the changes in cable length. For some values of cable length large changes are observed in all amplitudes: fundamental, dc, and harmonics. At other lengths the amplitudes are relatively insensitive. The data suggest that at some cable lengths the cable impedance is a dominant part of the total impedance of the external circuit, while it is insignificant at other cable lengths, but a complete understanding of the data, explaining the connection between the external circuit impedance and the plasma harmonics, is not currently available.

One aspect of the interaction between the nonlinear plasma impedance and the impedance of the external circuit has been investigated in detail: the generation of subharmonics. Subharmonics at half the fundamental frequency have been observed in the GEC cell [10] and models have been developed to determine the conditions on the discharge non linearity and external circuit that are necessary to generate them. Nevertheless, subharmonics have only been reported in this one study, in which the external circuit was purposely modified in a deliberate attempt to generate them. It seems that the conditions necessary for generation of the subharmonics are not easily or frequently attained in the GEC cell, for typical discharges and typical external circuits.

One means of reducing the sensitivity of the discharge to the external circuit has been demonstrated [11]. A filter, designed to have a high transmission at the fundamental frequency, but low transmission at the harmonics, can be placed on the cable that powers the cell. The filter serves to isolate harmonics generated in the discharge from all portions of the external network upstream of the filter, including the rf source and matching network. Using the filter, a high degree of reproducibility can be obtained between cells powered by different equipment [11]. Of course, to assure the greatest reproducibility it is still necessary that the portion of the network between the filter and the cell be electrically identical. This would include assuring identical values for all of the cell parasitics—not just in the powered electrode, but in the ground electrode as well. Indeed, it has been observed that the harmonic currents flowing through the ground electrode are quite sensitive to the ground electrode parasitics [6].

### 4.2 Gas Impurities

**Fig. 6:**
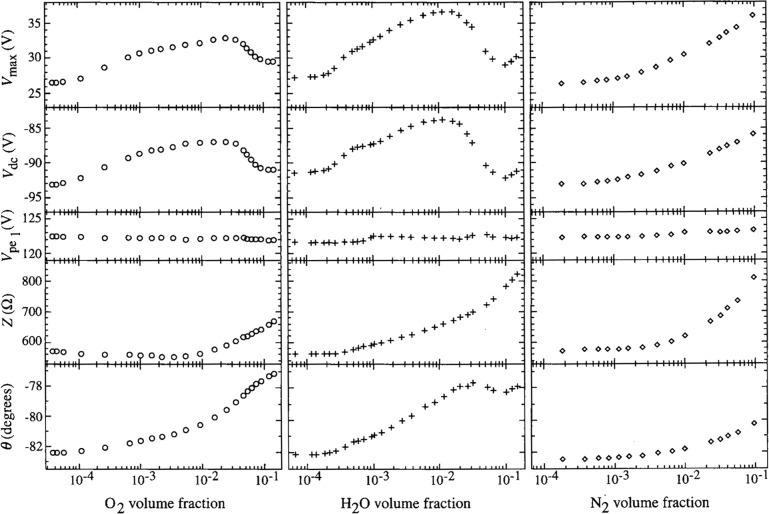
Electrical data for Ar/O_2_ (left), Ar/H_2_O (middle), and Ar/N_2_ (right) plasmas at 13.3 Pa (100 mTorr), indicating the sensitivity of argon discharges to impurity gases. The volume fraction given on the *x*-axes were obtained from calibrated mass spectrometer measurements. The electrical parameters on the *y*-axes include: *V*_max_, the maximum value attained by the corrected voltage waveform *V*_pe_(*t*); *V*_dc_ and $V_{{\text{pe}}_{1}}$, the dc and fundamental (13.56 MHz) components of *V*_pe_(*t*); and the magnitude (*Z*) and phase (*θ*) of the impedance $V_{{\text{pe}}_{1}}/I_{{\text{pe}}_{1}}$, where $I_{{\text{pe}}_{1}}$ is the fundamental current component. (From Ref. [13].)

The comparisons shown in Figs. 2 to 4 were performed using cylinders of argon gas purified to better than 99.999 %. However, gas-phase impurities in the cell during plasma operation could have been much higher. Leaks, backstreaming from pumps, desorption or sputtering of species from surfaces, and malfunctioning of mass flow controllers can all contribute significant levels of gas-phase impurities. Furthermore, discharges in argon, an electropositive gas, are expected to be very sensitive to the addition of minute quantities of electronegative gases [12]. To determine if gas-phase contamination represents an important source of irreproducibility, mixtures of argon with oxygen, nitrogen, and water, three common contaminants in plasma systems, have been investigated [13]. Results are shown in Fig. 6. Significant changes in the electrical parameters were observed at volume fractions as low as 6×l0^−5^ for O_2_, 2 × 10^−4^ for H_2_O, and 6 × 10^−4^ for N_2_. Presumably, the addition of each gas decreases the electron concentration in the plasma, partly by diluting the argon but more importantly because of attachment by electronegative species. Reduced electron concentration produces higher and more resistive plasma impedances. Changes in the electron concentration affect the impedance of different regions of the plasma to different degrees, producing changes in the plasma potential and the dc bias. It is not clear if these results can be generalized to other gases, but in any case, to assure the greatest reproducibility, good vacuum practice should be followed to minimize the background level of any impurities.

### 4.3 Surface Conditions

Discharges can also be affected by changes at the electrode and chamber surfaces that are in contact with the plasma. These surfaces, bombarded by ions and electrons from the plasma, emit secondary electrons that can serve to sustain or intensify the discharge. The secondary electron yield varies widely for different materials, so differences in cell materials may represent another source of irreproducibility among cells. The GEC cell was originally designed with aluminum electrodes, with the chamber and ground shields made of stainless steel. Some cells, however, have been constructed with stainless steel electrodes. Current and voltage measurements in these cells appear to differ from cells with aluminum electrodes, but is has not yet been proven that the differences result from the different electrode materials. In another cell, the standard ground electrode was replaced by a mass spectrometer that could be equipped with an end-plate of either stainless steel or aluminum [14]. In that cell, electrical data did not exhibit a significant dependence on the choice of material, for Ar, He, N_2_, and O_2_ discharges. The composition of the powered electrode was not varied in that study, so secondary electron effects at the powered electrode can not be ruled out.

**Fig. 7:**
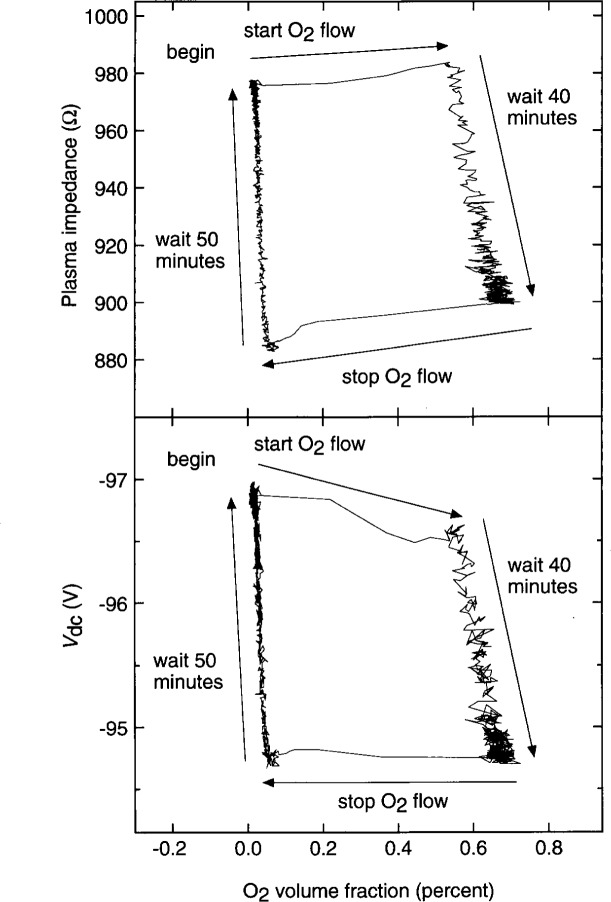
The magnitude of the plasma impedance $V_{{\text{pe}}_{1}}/I_{{\text{pe}}_{1}}$, and the dc component of the voltage on the powered electrode *V*_dc_, for Ar/O_2_ mixtures at 2.7 Pa (20 mTorr), vs the O_2_ volume fraction, obtained from calibrated mass spectrometer measurements. In the experiment, a constant O_2_ flow was turned on and then shut off 40 min later. The hysteresis in the data arises from the slow formation of an oxygen-rich layer on the surface of the aluminum electrodes, and a subsequent slow removal of the layer by sputtering. Data from Ref. [13].

Secondary electron yields are extremely surface-sensitive, responding to adsorption or reactions that occur over the topmost atomic monolayer(s) of a surface [15]. Thus, even if the electrode materials are not varied, changes in plasma characteristics can be produced by more subtle changes occurring on the surfaces. Such effects are particularly relevant for aluminum electrodes. When aluminum metal is exposed to oxidizing environments, a native oxide forms, and secondary electron yields are are significantly higher for oxidized aluminum [16]. In one GEC cell with aluminum electrodes, hysteresis was observed when the electrical parameters of Ar/O_2_ discharges were measured as a function of the gas-phase concentration of O_2_ [13]. In one experiment, shown in Fig. 7, a slow decrease in plasma impedance was observed for oxygen-rich conditions, consistent with a slow increase in secondary electron emission due to the build-up of an oxygen-rich surface layer. The change in plasma impedance was reversed in oxygen-poor conditions as oxygen was removed from the surface presumably by Ar sputtering. Trace quantities of oxygen, present in any cell, can thus significantly affect the discharge, whether the oxygen is in the gas phase (where it causes the plasma impedance to increase) or on surfaces (causing it to decrease). Other surface processes may cause similar changes in the electrical characteristics, especially in etching and deposition plasmas. Further study is required to identify and characterize them.

## 5. Comparisons with Other Measurements

Although current and voltage measurements can easily detect changes in plasma conditions in one cell or differences between cells, it is nevertheless often difficult to interpret the measurements. The current and voltage characteristics measured at the powered electrode include contributions from all regions of the plasma and all of its sheaths, and each of these may have a different, complicated relation between current and voltage. A single pair of current and voltage waveforms does not provide enough information to unambiguously characterize the electrical properties of each sheath. By employing additional electrical measurements, however, one can obtain a less ambiguous characterization of the discharge electrical properties and better understanding of their origin. For example, in one study [17], in addition to the measurements at the powered electrode, current was measured at the grounded electrode and at the ground shields (by mounting inductive d*I/*d*t* current probes inside the cell), to determine how the current flowing into the plasma through the powered electrode distributed itself among the different grounded surfaces in the cell. Typically, for argon discharges, most of the current was found to flow to the chamber wall. Another study combined the powered electrode mea surements with measurement of the current at the ground electrode and the rf voltage on a wire inserted in the plasma, to determine how the rf voltage as well as the current and impedance were divided among the different sheaths of the plasma [6]. Under most conditions in argon, the largest fraction of the applied voltage is dropped across the sheath at the powered electrode, hence this sheath makes the dominant contribution to the overall impedance of the discharge.

Additional understanding can be obtained by comparing electrical data with the results of other plasma diagnostic techniques. Electrical measurements have been performed simultaneously with measurements of the ion kinetic energy distributions at the grounded electrode [13,18]. Similar trends are often observed—when electrical data indicate changes in the voltage drop across the sheath at the grounded electrode, corresponding changes are observed in the ion energies. Similar trends are often observed in optical emission data—changes in the optical emission spatial profile caused by the onset of emission at the ground electrode or in the bulk occur at conditions where electrical data suggest increases in the current density and electric fields there [19]. Qualitatively, the behavior of sheath widths measured by optical emission agree with electrical sheath widths defined by sheath capacitance values determined from measurements of the fundamental current and voltage [20].

**Fig. 8:**
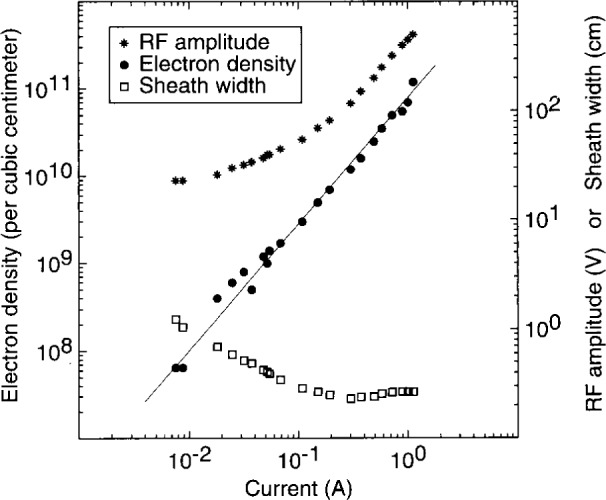
Comparison of current and voltage data with electron densities measured by microwave interferometry, for argon discharges at 66 Pa (500 mTorr) in a GEC cell (from Ref. [9]). Also shown is the electrical sheath width *s*, calculated from *C = ϵ*_O_*A/s* and Im(*Z*)= −l/(*ωC*), where *Z* is the discharge impedance defined by the *V*_pe_(*t*) and *I*_pe_(*t*) components at the fundamental frequency *ω*/2π= 13.56 MHz. *ϵ*_O_ is the permittivity of vacuum, 8.85 × 10^−14^ F/cm, and *A* is the electrode area.

**Fig. 9:**
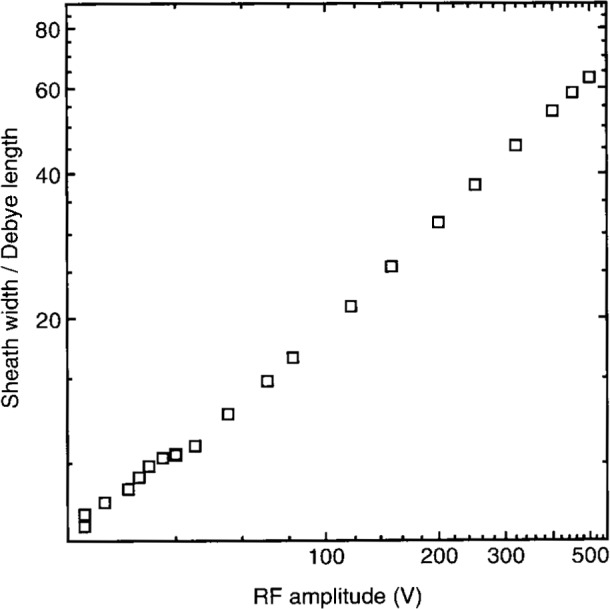
The electrical sheath width, normalized by the Debye length, to remove the explicit dependence of sheath width on electron density and reveal the explicit dependence of the electrical sheath width on rf voltage. The data is from Fig. 8, originally from Ref. [9]. The Debye length was calculated using a constant value of 3 eV for the electron temperature [9].

Perhaps the most relevant plasma parameter for comparison to the electrical data is the electron concentration *n*_e_. A microwave interferometer [9,21] and a Langmuir probe [21] have been used to measure *n*_e_ in the GEC cell, in conjunction with electrical measurements. One set of results for argon plasmas at 66 Pa (500 mTorr) is shown in Fig. 8. The capacitance of the discharge is dominated by the sheath at the powered electrode [6], so the electrical width of this sheath can be calculated, as shown. Together, the data in Fig. 8 are particularly useful for testing models of the electrical behavior of rf plasma sheaths. These models [22,23,24] predict that the electrical sheath width should be equal to the Debye length (in the plasma just outside the sheath) times some function of the rf voltage across the sheath. By measuring *n*_e_ (and the electron temperature *T*_e_) the dependence of the sheath width on Debye length can be factored out, and the explicit dependence of the sheath width on sheath voltage (and pressure) can be determined. Figure 9 shows the result of such an analysis, based on the electrical data and *n*_e_ values of Fig. 8. Results such as those shown in Fig. 9 can be directly compared to model predictions, facilitating experimental testing of the models. Confirmation of the models, or development of more accurate models should present models be found to be invalid, would greatly increase our ability to interpret experimental current-voltage data.

## 6. Conclusions

Measurements of current and voltage waveforms are a useful means of assessing the reproducibility of discharges in GEC cells, provided that the many possible sources of error in these measurements are minimized. To obtain the greatest degree of reproducibility between cells requires that the electrical networks, gas impurities, and interior surfaces of the cells be identical. To some extent, current and voltage data can be interpreted and changes in the data can be used to infer how fundamental properties of the plasma are changing, but further work is needed to obtain a complete understanding of the data.

## Footnotes

1 Certain commercial equipment, instruments, or materials are identified in this paper to foster understanding. Such identification does not imply recommendation or endorsement by the National Institute of Standards and Technology, nor does it imply that the materials or equipment identified are necessarily the best available for the purpose.

## References

[1] Hargis PJ, Greenberg KE, Miller PA, Gerardo JB, Torczynski JR, Riley ME, Hebner GA, Roberts JR, Olthoff JK, Whetstone JR, Van Brunt RJ, Sobolewski MA, Anderson HM, Splichal MP, Mock JL, Bletzinger P, Garscadden A, Gottscho RA, Selwyn G, Dalvie M, Heidenreich JE, Butterbaugh JW, Brake ML, Passow ML, Pender J, Lujan A, Elta ME, Graves DB, Sawin HH, Kushner MJ, Verdeyen JT, Horwath R, Turner TR (1994). The Gaseous Electronics Conference Radio-Frequency Reference Cell: A defined parallel-plate radio-frequency system for experimental and theoretical studies of plasma-processing discharges. Rev Sci Inst.

[2] Greenberg KE, Hargis PJ, Miller PA (1990). The GEC Reference Cell: Diagnostic Techniques and Initial Results.

[3] Miller PA (1991). Electrical characterization of rf plasmas. Proc SPIE.

[4] Sobolewski MA (1992). Electrical characterization of radio-frequency discharges in the GEC Reference Cell. J Vac Sci Technol.

[5] Verdeyen JT (1992). Measurements and Analysis of the Equivalent Circuit of the GEC RF Reference Cell, SAND92-7284.

[6] Sobolewski MA (1995). Electrical characteristics of argon radio-frequency glow discharges in an asymmetic cell. IEEE Trans Plasma Sci.

[7] Miller PA, Kamon M (1990). Electrical Characterization of the GEC Reference Cell.

[8] Sobolewski MA, Whetstone JR (1992). Electrical measurements for monitoring and control of rf plasma processing. Proc SPIE.

[9] Overzet LJ, Leong-Rousey FY (1994). Time resolved power measurements to pulsed discharges in the Gaseous Electronics Conference reference reactor. Plasma Sources Sci Technol.

[10] Miller PA, Romero LA, Pochan PD (1993). Subharmonics and rf-plasma sheaths. Phys Rev Lett.

[11] Miller PA, Anderson H, Splichal MP (1992). Electrical isolation of radio-frequency plasma discharges. J Appl Phys.

[12] Paranjpe AP, McVittie JP, Self SA (1990). Scaling laws for radio frequency glow discharges for dry etching. J Vac Sci Technol.

[13] Sobolewski MA, Olthoff JK (1994). Electrical Sensors for Monitoring RF Plasma Sheaths. Proc SPIE.

[14] Olthoff JK, Van Brunt RJ, Radovanov SB Effect of electrode material on measured ion energy distributions in radio-frequency discharges. Appl Phys Lett.

[15] Halbritter J (1992). On conditioning: Reduction of secondary and rf-field emission by electron, photon, or helium impact. J Appl Phys.

[16] Lewis MA, Glocker DA, Jorne J (1989). Measurements of secondary electron emission in reactive sputtering of aluminum and titanium nitride. J Vac Sci Technol.

[17] Miller PA (1992). Dependence on excitation symmetry of electrical parameters and radial currents in a parallel-plate RF discharge.

[18] Olthoff JK, Van Brunt RJ, Sobolewski MA, Williams WT (1992). Ion kinetic-energy distributions and electrical measurements in argon-oxygen rf glow discharges. Proc Tenth Intl Conf on Glow Discharges and Their Applications.

[19] McMillin B 2-D laser-induced fluorescence imaging of metastable density in low-pressure rf argon plasmas with added O_2_, Cl_2_ or CF_4_. J Appl Phys.

[20] Djurovic S, Sobolewski MA

[21] Overzet LJ, Hopkins MB (1993). Comparison of electron-density measurements using a Langmuir probe and microwave interferometer in the Gaseous Electronics Conference reference reactor. J Appl Phys.

[22] Lieberman MA (1988). Analytical solution for capacitive rf sheath. IEEE Trans Plasma Sci.

[23] Lieberman MA (1989). Dynamics of a Collisional, Capacitive RF Sheath. IEEE Trans Plasma Sci.

[24] Godyak VA, Sternberg N (1990). Dynamic model of the electrode sheaths in symmetrically driven rf discharges. Phys Rev A.

